# A Mahar Quarter

**Published:** 1897-11

**Authors:** 


### A MAHAR QUARTER.

The Maharwada in Mungulwar Peth is a collection of houses, only fifty-four, skirting a crowded district, placed upon a triangular knoll, and sloping down to the Nagzari nullah, which converts it into a small peninsula. The spot is a couple of hundred yards from the G. I. P. overbridge near the Sangam. I will take these pest spots one by one. The Nagzari nullah takes a curve through the dirtiest part of the town and its sluggish waters receive a considerable contribution of sewage from the Peshwa's system. As it approaches its embrechure into the Poona river it makes a sharp turn, half enclosing the Mahar quarter. Here, having passed through the town, it is indescribably filthy, and owing to the curve its flow slackens, and a sediment of sewage falls like a bed along its borders. The Mahar houses fringe this bed. The stench from the nullah alone streaming round their quarters would overwhelm any people but the Mahars. Adjoining the nullah is a latrine over which, as over the stream, westerly breezes all the hot weather evenings blow straight into their dwellings. The latrine was uncared for and filthy in the last degree. Along the tiny foreshore, not into the nullah, but on its banks the sullage of eight undrained peths was daily poured from carts and mussacks. The liquid ordure and various liquid waste of nearly 38,000 people was daily poured out on the land at the very door of these unfortunate people. On the northeast is another public latrine as foul as the other and just as close. A burial-ground adjoins this latrine. The condition of this burial-ground was the most disgraceful exhibition of neglect that crossed my entire experience. The dead are buried thick as leaves in Valhambrosa, many of them scarcely a foot from the surface, and one of the oddest sights from the bridge is to see the little hill dotted all over with lilliputian headstones no bigger than a child's hand. On one side of the stream the pariah dogs and probably jackals, have eaten away the side of the hillock, revealing skeleton forms and bones embedded a foot or so below the surface. Along the side of the nullah and all over the surface were scattered skulls and bones in promiscuous confusion mixed with ashes, rags and refuse such as only Mahars reject. In parts the ground had collapsed where rooting things had undermined it, and the bones stuck out in fantastic weirdness on all sides. This small spot is a populous quarter, at once a charnel-house and refuse depot, had no appearance of ever having been cleaned. Thirty plague deaths occurred among this little group of houses. The dead were buried in the small knoll. The soil was poisoned with plague germs, yet old women dug away the earth in the light of day from under the graves. There was quite a run on this fashionable commodity, and it sold for two pice a gamela because it made such capital loam with which to *leep* the floors.—*Indian Medical Lancet.*

